# The kinesin-13 KLP10A motor regulates oocyte spindle length and affects EB1 binding without altering microtubule growth rates

**DOI:** 10.1242/bio.20148276

**Published:** 2014-06-06

**Authors:** Kevin K. Do, Kim Liên Hoàng, Sharyn A. Endow

**Affiliations:** Department of Cell Biology, Duke University Medical Center, Durham, North Carolina 27710, USA

**Keywords:** Kinesin-13, KLP10A, Depolymerizing motor, Spindle, Microtubule dynamics, EB1, *Drosophila*

## Abstract

Kinesin-13 motors are unusual in that they do not walk along microtubules, but instead diffuse to the ends, where they remove tubulin dimers, regulating microtubule dynamics. Here we show that *Drosophila* kinesin-13 *klp10A* regulates oocyte meiosis I spindle length and is haplo-insufficient – KLP10A, reduced by RNAi or a loss-of-function P element insertion mutant, results in elongated and mispositioned oocyte spindles, and abnormal cortical microtubule asters and aggregates. KLP10A knockdown by RNAi does not significantly affect microtubule growth rates in oocyte spindles, but, unexpectedly, EB1 binding and unbinding are slowed, suggesting a previously unobserved role for kinesin-13 in mediating EB1 binding interactions with microtubules. Kinesin-13 may regulate spindle length both by disassembling subunits from microtubule ends and facilitating EB1 binding to plus ends. We also observe an increased number of paused microtubules in *klp10A RNAi* knockdown spindles, consistent with a reduced frequency of microtubule catastrophes. Overall, our findings indicate that reduced kinesin-13 decreases microtubule disassembly rates and affects EB1 interactions with microtubules, rather than altering microtubule growth rates, causing spindles to elongate and abnormal cortical microtubule asters and aggregates to form.

## INTRODUCTION

The kinesin motor proteins bind to microtubules and hydrolyze ATP, producing force in the cell to transport cargo along microtubules or slide microtubules relative to one another. The motors couple cellular regions to one another via the microtubule cytoskeleton, a feature common to eukaryotic cells that forms a network in the cell. Many kinesin motor proteins play essential roles in spindle assembly and elongation in dividing cells, driving spindle and chromosome motility during mitosis and meiosis (Mitchison and Salmon, 2001; Mogilner and Craig, 2010). The motors crosslink and slide microtubules, producing force to assemble spindles, and position chromosomes for movement to opposite poles.

Although most kinesins move directionally along microtubules towards the plus or minus end, motors in the kinesin-13 subfamily are unusual in that they bind to microtubules and diffuse to the ends, where they promote microtubule disassembly, rather than walking along the lattice (Walczak et al., 1996; Desai et al., 1999). These motors also differ from other kinesin proteins in that they have a centrally located motor domain (Wordeman and Mitchison, 1995) and are associated with the centromeres of mitotic and meiotic chromosomes (Wordeman and Mitchison, 1995; Zou et al., 2008; Vogt et al., 2010). The kinesin-13 motors have attracted considerable interest because of their unusual activity in disassembling microtubules and regulating microtubule dynamics, and also because of the possibility that they underlie or contribute to poleward chromosome movement in anaphase.

Humans have three kinesin-13 homologues, each of which is thought to have somewhat different but overlapping functions in mitosis (Manning et al., 2007; Ems-McClung and Walczak, 2010). The motors are involved in spindle assembly and regulation of spindle microtubule dynamics, and kinetochore-to-microtubule attachments. Because of these roles, they are essential for chromosome segregation. *Xenopus* has two kinesin-13 motors that have been shown to have differing roles in *in vitro* extract spindles – one regulates dynamic instability and controls spindle length, whereas the other is nonessential for spindle assembly (Ohi et al., 2007). *Drosophila*, like humans, encode three kinesin-13 motors, which have been interpreted to mediate chromosome-to-pole movement during anaphase of mitosis by microtubule disassembly at the kinetochore or pole (Rogers et al., 2004; Rath et al., 2009).

Although the kinesin-13 motors have been well studied in mitotically dividing cells, only a few studies exist of these motors in meiosis. KLP10A, one of the *Drosophila* kinesin-13 motors, has been implicated by *RNAi* knockdown and mutant analysis in meiotic spindle length regulation in oocytes (Zou et al., 2008; Radford et al., 2012). Images of oocytes show KLP10A localized to the meiosis I (MI) spindle (Zou et al., 2008; Radford et al., 2012), as well as the spindle pole bodies and meiotic chromosome centromeres (Zou et al., 2008). KLP10A has also been observed bound to cortical microtubules attached to the spindle pole bodies, implying a role in anchoring the spindle to the cortex – fly lines expressing a dominant-negative *klp10A* mutant showed oocyte spindles that were not only abnormal in overall structure, but were oriented more vertically to the cortex than wild type (Zou et al., 2008).

Although previous studies have established that kinesin-13 plays a role in spindle length regulation, the mechanism by which this occurs is not certain. It has been suggested to involve an increase in microtubule catastrophes mediated by kinesin-13 motors (Walczak et al., 1996; Rogers et al., 2004). To shed light on this issue, we studied the effects of reduced KLP10A levels on oocyte spindles. The genetic and cytological effects in oocytes yield new information about KLP10A interactions with the MI spindle and cortical microtubules. Moreover, the effects of reduced KLP10A on microtubule growth in the spindle revealed by EB1 tracking and on EB1 binding interactions with spindle microtubules, as analyzed by fluorescence photobleaching assays, show a previously unobserved interaction of kinesin-13 with EB1 in the spindle.

## MATERIALS AND METHODS

### Stocks

Flies were raised on cornmeal agar medium at 22°C. The *klp10A RNAi* line, y^1^ sc* v^1^; *P{y^+t7.7^ v^+t1.8^ = TRiP.HMS00920}attP2* (Ni et al., 2011), was obtained from the Bloomington Drosophila Stock Center. The *klp10A RNAi* knockdown females express *dsRNA* for *klp10A RNAi* under *UAS* control driven by *P{matα4-GAL-VP16}V37* (Januschke et al., 2002; Dubin-Bar et al., 2011; Shapira et al., 2011), denoted here as *αtubGal4*. In some experiments, *P{GAL4-nos.NGT}40* was used as the *gal4* driver (Tracey et al., 2000). Expression of the *gal4* driver is known to be temperature-dependent with the lowest expression at 16°C and the greatest at 29°C (Duffy, 2002); the *klp10A RNAi* experiments reported here were performed at 22°C, which is expected to produce an intermediate *RNAi* knockdown effect. More severe conditions were not examined, given that the *RNAi* knockdown females were already sterile and produced oocytes with greatly elongated spindles and severe effects on cortical microtubules. A P-element induced loss-of-function mutant (Peter et al., 2002), *klp10A ThbA lines #11* and *#50*, referred to here as *klp10A P mut*, was a gift of Drs Helen Rangone and David M. Glover (Delgehyr et al., 2012). *klp10A P mut line #50* is more fertile than *line #11* and was used for most of the genetic and cytological tests. The *klp10A P mut* chromosome is maintained with the *FM7i P{ActGFP}* balancer chromosome. An *eb1-gfp* transgene, *M6F1* (Liang et al., 2009), was used to label oocyte spindles and cortical microtubules. Oocytes carrying *eb1-gfp* and wild type for *klp10A* are referred to throughout as wild type. Females carrying *w^1118^* as an *X* chromosome marker and wild type for *klp10A* were used as controls for genetic tests, and are again referred to as wild type.

### Genetic tests

Genetic tests to determine the effects of *klp10A* loss of function on female fertility were performed by mating *klp10A RNAi* knockdown females heterozygous for *klp10A RNAi* and the *αtubGal4* driver to *w^1118^/B^S^Y* males in single pairs and scoring the matings for offspring. Fertility tests of females heterozygous for *klp10A P mut* and *FM7i* or a normal sequence (Oregon R) *X* chromosome, together with tests of wild-type females, were similarly performed, except the crosses were transferred to new vials every 2–8 days and the dates on which offspring eclosed were recorded along with their phenotype. Plots of offspring produced over time by the *klp10A P mut* heterozygous and wild-type females were made by calculating running totals over time from the recorded data and averaging them over the number of crosses. Three of the six *klp10A P mut*/*FM7i* heterozygous females died during the test crosses. Tests of *klp10A P mut/Y* males for fertility were performed by crossing to *w^1118^* females and scoring matings for offspring.

Tests for effects of the *klp10A P mut* allele on *X* chromosome segregation were performed by scoring offspring of *klp10A P mut*/*FM7i* female and *w^1118^*/*B^S^Y* male single-pair matings. Meiotic *X* chromosome nondisjunction results in diplo- and nullo-*X* gametes, half of which are recovered as *X*/*X*/*B^S^Y* females and *X*/*0* males; meiotic *X* chromosome loss also gives rise to *X*/*0* males, increasing their frequency above that of the *X*/*X*/*B^S^Y* females (Komma et al., 1991). Early mitotic loss of the *X* chromosome, which gives *XX*/*X0* gynandromorphs, or chromosome *4* during meiosis or mitosis, producing Minute offspring, was also scored. Tests of *klp10A P mut*/*FM7i* females gave 3 putative *X*/*X*/*B^S^Y* females and 3 *X*/*0* males (abnormal offspring, *n* = 6; abnormal gametes, *n* = 12) among the offspring; the *X*/*0* males were attributed to nondisjunction because of the absence of gynandromorphs and Minutes, indicating that chromosome loss is highly infrequent; the exceptional females were scored as nondisjunctional from their phenotype and the expectation that an equal number of *X*/*X*/*B^S^Y* females should be recovered as nondisjunctional *X*/*0* males.

Statistical tests for significance of chromosome segregation data were performed by calculating the probability of observing *i* exceptional offspring, assuming that the offspring form a Poisson distribution, *p*(*i*) = (*e*^−m^_*_m^i^)/*i*!, where *m* = *#* expected; *m* was calculated assuming that the wild-type and mutant females being tested were from the same population (Komma et al., 1991). A χ^2^ test of significance was not used because the number of expected exceptional offspring was too low (<5) to give accurate results (Yates, 1934).

### Western blots

Wild-type (Oregon R), *klp10A RNAi* knockdown and *klp10A P mut/FM7i* females were fattened on yeast for three days, then ovaries were dissected in HEM100 (10 mM HEPES pH 7.2, 1 mM MgCl_2_, 1 mM EGTA, 100 mM NaCl) containing protease and phosphatase inhibitors, and homogenates were made. Western blots with lanes containing protein corresponding to one ovary were prepared, reacted with KLP10A-specific antibodies (a gift of Dr David Sharp), and developed using the alkaline phosphatase system (Promega Corp.). Reaction with actin antibodies (Sigma–Aldrich) was performed subsequently. Western blots were scanned and saved as digital images, and KLP10A protein levels were corrected for loading and quantified relative to wild type in ImageJ (W. S. Rasband, ImageJ, Bethesda, Maryland, USA (NIH), 1997–2012).

### Live imaging of oocytes

*klp10A RNAi* knockdown females expressing EB1-GFP (Liang et al., 2009) were produced by mating *eb1-gfp M6F1*; *TM3 Sb*/+ females to *klp10A RNAi* males. The *eb1-gfp M6F1*/+; *klp10A RNAi/TM3 Sb* female F_1_ offspring were mated to *eb1-gfp M6F1; αtubGal4*/*TM3 Sb* males to give non-*Sb klp10A RNAi*/*αtubGal4* knockdown female F_2_ offspring that were either homozygous or heterozygous for *eb1-gfp M6F1*. Oocytes of the non-*Sb* F_2_ females were analyzed by live imaging and used in FRAP assays. For analysis of spindles and cortical microtubules, oocytes were imaged at 18°C using a Bio-Rad Radiance2100 confocal scanhead mounted on a Zeiss Axioskop 2 microscope (Carl Zeiss, Thornwood, NY, USA), a Zeiss EC Plan-Neofluar 40×/1.3 NA oil immersion objective, Bio-Rad LaserSharp 2000 software, and the 488-nm line of a 10 mW Kr–Ar laser. Image analysis was performed in ImageJ.

### Spindle length measurements

Oocytes were classified as late stage 13 or stage 14 based on the presence (late stage 13) or absence (stage 14) of a nurse cell cap associated with the dorsal appendages (Spradling, 1993). MI spindles were measured in ImageJ from Z-series projections after live imaging. Spindle lengths were corrected for the tilt of the spindle relative to the oocyte cortex by assuming that they form the hypotenuse of a right triangle, so that spindle length, 
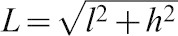
, where *l* is the spindle length measured from the projected images and *h* is the spindle height, equal to the distance between the two poles (Zou et al., 2008). Error estimates are given as ± s.d., calculated from the spindle length measurements after correction for the tilt of the spindle. Tests of significance for spindle length differences were performed using Student's *t*-test with unequal variances, also known as Welch's *t*-test (Welch, 1947).

### EB1 particle tracking

Particle tracking on late stage 13 wild-type and *klp10A RNAi* knockdown oocyte spindles was performed using ImageJ. The images were first contrast-enhanced by applying a Gaussian blur (radius = 2 pixel), subtracting the blurred sequence from the original image sequence, then adding the difference back to the original image sequence. EB1 particles in the contrast-enhanced sequence were tracked using the Manual Tracking macro in FIJI (Schindelin et al., 2012). The EB1 tracks were assigned as moving towards the equator or pole, and the mean velocity was calculated from the distance and time. Images (60 frames/stack) were acquired at a spatial resolution of 0.100 µm/pixel and time resolution of 1 s/frame. Velocities are given as the mean ± s.e.m. Microtubule plus ends were counted as paused if they moved <1 pixel (i.e. <0.100 µm) over ≥1 s. Statistical tests of significance for velocity differences were evaluated using Student's *t*-test with unequal variances; tests of significance for differences in the frequency of paused EB1 particles were performed using a χ^2^ test. Possible effects of spindle movement during imaging on calculated particle velocities were analyzed and found to be negligible; corrections for spindle movement were therefore not included in the final data analysis.

### Photobleaching assays

FRAP assays were performed essentially as described (Hallen et al., 2008). Briefly, late stage 13 oocyte MI spindles were imaged at 22°C on a Zeiss LSM 780 confocal microscope (Carl Zeiss, Thornwood, NY, USA) using ZEN software, a Zeiss Plan-Apochromat 40×/1.4 NA DIC oil immersion objective, and the 488 nm line of a 25 mW Ar laser at >50% power. Three prebleach images were recorded, followed by 4 bleach scans in an ROI of radius *w* = 1 or 0.5 µm, then two hundred and ninety-seven recovery images were collected at ∼122 ms time intervals. The mean pixel intensity of the ROIs before and after bleaching was measured in ImageJ. The time stamps for the recovery images were set to *t* = 0 at the end of the last bleach, recorded in the file metadata. Data were normalized to the mean fluorescence of the last 180 data points and corrected for loss during recovery imaging by adding back the fluorescence lost from an adjacent unbleached ROI. The first 122 data points, which accounted for >98% of the recovery, were weighted by averaging every 5 time points after the first 122 points and entering the averages as single points (Hallen et al., 2008). Data from the assays were averaged, plotted versus time, and fit to kinetic models for fluorescence recovery (Sprague et al., 2004) using Kaleidagraph (Synergy Software, Reading, PA, USA) and MATLAB (The MathWorks Inc., Natick, MA, USA). Large and small ROI data were fit concurrently to the models (Hallen et al., 2008).

Fits of the full model for fluorescence recovery of Sprague et al. (Sprague et al., 2004), which accounts for all possible modes of recovery by a single binding reaction in the presence of diffusion, were performed using the inverse Laplace transform given in equation 8 of Hallen et al. (Hallen et al., 2008). The fits yielded kinetic parameters that led us to try reaction-dominant models, based on criteria explained in the Results. The single-state reaction-dominant model, 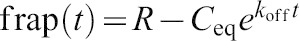, was tried first, where *R* is the equilibrium fluorescence intensity after recovery, *C*_eq_ is the equilibrium fluorescence due to binding, and *k*_off_ is the dissociation rate constant (Sprague et al., 2004). This model assumes that diffusion occurs very rapidly and recovery is dominated by the kinetics of a single binding state. Because the residuals showed an obvious trend, indicating that the single-state reaction-dominant model was insufficient to account completely for the kinetics of the recovery, we then tried a two-state reaction-dominant model, 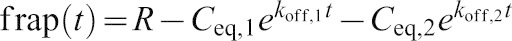 (Sprague et al., 2004). The unbound protein fluorescence is given by *F*_eq_ = 1−*C*_eq_ and the pseudo first-order binding constant by 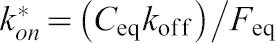. Error estimates were calculated from the parameter covariance matrix given by the MATLAB routine *leasqr.m* (R. Shrager, A. Jutan and R. Muzic, *leasqr.m*, 1994) (Hallen et al., 2008). The kinetic parameters that we report in Table 1 are from the two-state reaction-dominant model, which fit the data best. Fits of the data to the full model (Sprague et al., 2004) gave kinetic parameters that were consistent with the conclusion from the two-state reaction-dominant model that EB1 turnover is slower in the *klp10A RNAi* knockdown spindles than wild type.

**Table 1.**
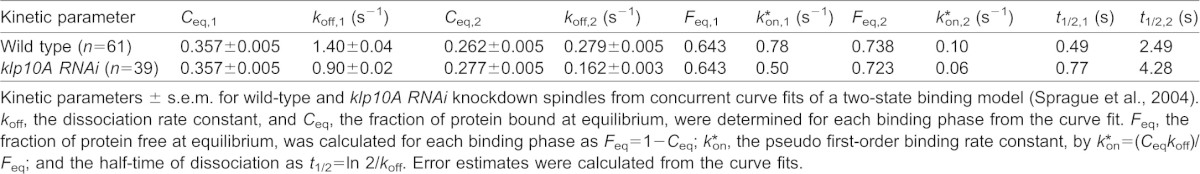
EB1-GFP FRAP kinetic parameters

## RESULTS

Naturally occurring *klp10A* mutants have not yet been reported, but we were able to obtain two lines that express reduced KLP10A levels to analyze the effects of *klp10A* loss of function in oocytes. The first is a recently released *klp10A RNAi* line (Ni et al., 2011) that can be induced in oocytes using the *αtubGal4* driver (Brand and Perrimon, 1993) and the second is a *P* element-induced loss-of-function mutant (Delgehyr et al., 2012). We first tested the two lines genetically for fertility. Because *klp10A RNAi* females carrying the *αtubGal4* driver produced no offspring, only the *klp10A P mut* line was tested for its effects on chromosome segregation in oocytes.

### *klp10A* affects female fertility

The *klp10A* knockdown and mutant lines were kept at 22°C, where the knockdown effects induced by *gal4* drivers are expected to be less severe than at higher temperatures (Duffy, 2002). Fertility tests of females heterozygous for *klp10A RNAi* and the *αtubGal4* driver, referred to here as *klp10A RNAi* knockdown females, gave no offspring (*n* = 19). The females laid many eggs but no larvae were observed in the vials, indicating high embryo lethality. As a control, females carrying either *klp10A RNAi* or *αtubGal4*, but not both, were tested and found to be fertile (*n* = 3), consistent with the observation that the *klp10A RNAi* and *αtubGal4* stocks are both fertile.

**Fig. 1.**
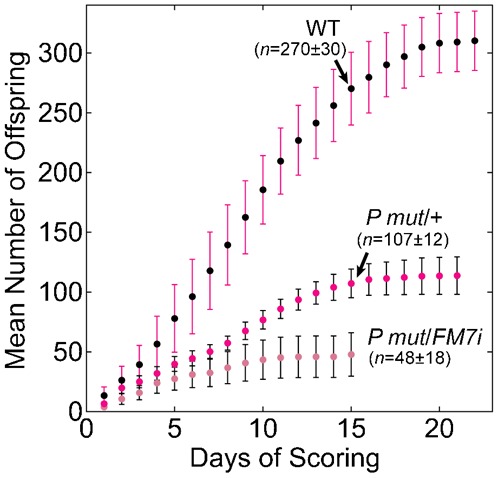
Fertility tests of *klp10A P mut*/+ heterozygous females. Mean running total offspring produced over time by *klp10A P mut* females carrying *FM7i* (*P mut*/*FM7i*; pink circles) or an Oregon R *X* chromosome (*P mut*/+; magenta circles), compared to wild type (WT; black circles). Error estimates for the mutant and wild-type populations are given as s.e.m.; the mean numbers of offspring on the final day of scoring for the *klp10A P mut*/*FM7i* crosses are shown for comparison.

The *klp10A P mut* allele is maintained with the *FM7i* balancer chromosome and was analyzed in heterozygous females. This mutant allele has been shown previously to express reduced levels of KLP10A (Delgehyr et al., 2012). Homozygous females are not observed in the stock and are presumed to be inviable (see below). *klp10A P mut*/*Y* males are present in the stock, but were sterile in tests for fertility (lines #11 and #50, *n* = 11), as reported previously (Delgehyr et al., 2012). Females heterozygous for the *klp10A P mut line #50* and *FM7i* balancer chromosomes showed variable fertility – of six single-pair matings, three were fertile, producing ∼90 offspring each; one was moderately fertile, giving 19 offspring; and two were essentially sterile with only two offspring each. On average, the fertile *klp10A P mut/FM7i* females each produced 88±4 offspring (mean ± s.d., *n* = 3); the overall average for the *klp10A P mut/FM7i* heterozygous females was 48±44 offspring (*n* = 6). By contrast, single-pair matings of wild-type females showed good fertility (310±50 offspring/mating, *n* = 4). Plots of the number of offspring produced by the matings over time showed a large difference for the *klp10A P mut/FM7i* females compared to wild type (Fig. 1). *klp10A P mut*/+ females carrying a normal sequence (Oregon R) *X* chromosome also produced significantly lower numbers of offspring (114±52 offspring/mating, *n* = 11) than wild type (Fig. 1), indicating that the reduced fertility is caused by *klp10A P mut*. The reduced fertility of the *klp10A P mut*/+ heterozygous females indicates that *klp10A* is haplo-insufficient.

Tests of *klp10A P mut line #50* heterozygous females carrying *FM7i* for chromosome segregation showed a small number of exceptional offspring (*n* = 12 gametes, total = 293; frequency, *ν* = 0.041), which were attributed to *X* chromosome nondisjunction during oocyte meiosis; loss of the *X* or chromosome *4*, giving gynandromorphs or Minute offspring, respectively, was not observed. The frequency of meiotic nondisjunction produced by *klp10A P mut*/*FM7i* females is significantly increased (*P* = 0.0005) relative to wild type (*n* = 2 gametes, total = 1242, *ν* = 0.00161). Nondisjunction is therefore expected to occur in the *klp10A P mut* stock and should result in a low level of *X/X/Y* females, which would be easily recognizable by their round (non-Bar) orange eyes. The absence of homozygous *klp10A P mut* females in the stock implies either that they are inviable or that nondisjunction occurs only in meiosis I.

### Reduced KLP10A results in longer oocyte spindles

**Fig. 2.**
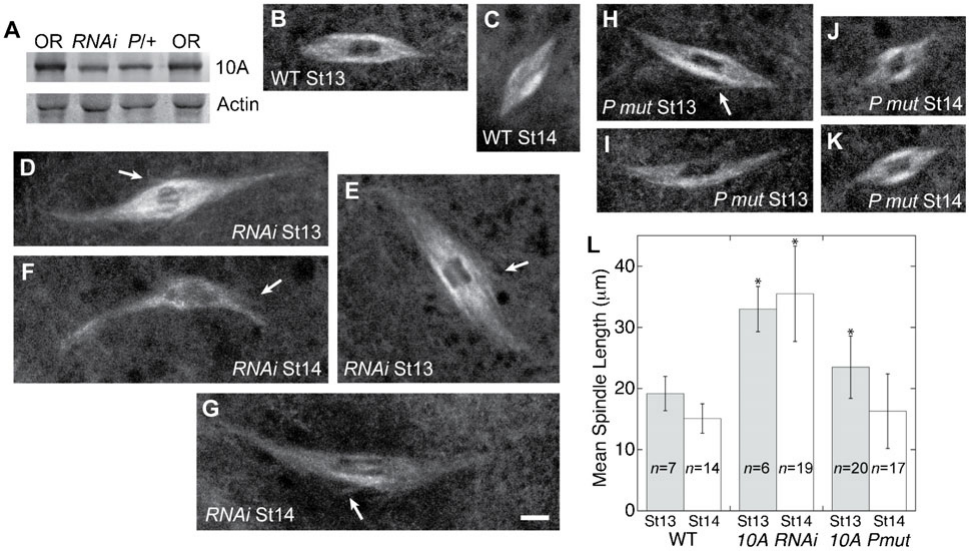
*klp10A RNAi* knockdown and *klp10A P mut* oocyte MI spindles. (A) KLP10A levels in ovaries. Western blot of *klp10A RNAi/αtubGal4* knockdown (*RNAi*) and *klp10A P mut/FM7i* (*P*/+) ovaries compared to wild type (Oregon R, OR), cross-reacted with KLP10A antibodies (10A, ∼110 kDa). Actin (∼45 kDa) is shown as a loading control. KLP10A levels, after correction for loading, are ∼45% and ∼60% of wild type in *RNAi* and *P mut*/+ ovaries, respectively. (B,C) Wild-type *eb1-gfp* oocyte MI spindles have well-defined poles, but differ in length between late stage 13 (B) and stage 14 (C). (D–G) *klp10A RNAi* knockdown oocyte spindles labeled with EB1-GFP are misshapen and frayed (arrows) with narrow, elongated poles. (H–K) *klp10A P mut*/+ late stage 13 oocyte spindles labeled with EB1-GFP have narrow poles, like *klp10A RNAi* knockdown spindles, but are less elongated; stage 14 spindles appear similar to wild type. (L) Histogram showing spindle lengths for wild-type (WT), *klp10A RNAi* knockdown (*10A RNAi*), and *klp10A P mut*/+ (*10A Pmut*) oocytes. Mean ± s.d. St13, late stage 13; St14, stage 14. Asterisks indicate significant differences relative to wild type of the same stage (*P*<0.05). Scale bar: 3 µm.

Western blots showed reduced KLP10A levels in ovaries of *klp10A RNAi* knockdown females (Fig. 2A). Despite the fact that the females were sterile, KLP10A levels were not fully reduced compared to wild type, but were easily detectable. Quantitation of the protein bands after correction for loading showed that KLP10A levels in the *klp10A RNAi* knockdown ovaries (females, *n* = 7) were ∼45% of wild-type levels (Oregon R; females, *n* = 4), consistent with our genetic data for *klp10A P mut*/+ female fertility and chromosome segregation that *klp10A* is haplo-insufficient. The reduced KLP10A levels that we observe are dependent on the *αtubGal4* driver, since ovaries from *klp10A RNAi* females expressing the *P{GAL4-nos.NGT}40* driver (Tracey et al., 2000) did not show reduced KLP10A levels by Western blot analysis (females, *n* = 11). Western blot analysis of *klp10A P mut line #50*/+ (*FM7i*) ovaries (females, *n* = 2) showed KLP10A levels that were ∼60% of wild type (Fig. 2A).

We examined live oocytes from wild-type and *klp10A RNAi* knockdown females, and *klp10A P mut line #50*/+ heterozygous females to determine the effects of reduced KLP10A levels on oocyte spindles. Wild-type oocyte MI spindles are bipolar with well-defined poles and a characteristic shape and length that differ between late stage 13, in which the MI spindle completes assembly, and stage 14, in which the mature, metaphase-arrested MI spindle is present (Fig. 2B,C). Late stage 13 wild-type spindles are bipolar, but longer (mean ± s.d., 19.2±2.8 µm, *n* = 7) (Fig. 2B,L) than the shorter and more compact stage 14 spindles (15.1±2.4 µm, *n* = 14) (Fig. 2C,L). By comparison, *klp10A RNAi* knockdown spindles in both late stage 13 (33.0±3.7 µm, *n* = 6) and stage 14 (35.5±7.8 µm, *n* = 19) displayed highly elongated or narrow poles and were frequently frayed, multipolar, or split (*n* = 29, total = 58, *ν* = 0.50) (Fig. 2D–G). Overall, the *klp10A RNAi* knockdown spindles were significantly longer than wild type in both stage 13 and 14 (*P*<0.0001 for each stage) (Fig. 2L), indicating severe effects in the late stages of spindle assembly that may prolong stage 13 and extend into stage 14.

Abnormal spindles were also observed in *klp10A P mut*/+ heterozygous oocytes, again indicating *klp10A* haplo-insufficiency, but the spindle defects were not as severe as those in *klp10A RNAi* knockdown oocytes (Fig. 2H–K). The frequency of abnormal spindles in late stage 13 oocytes (*n* = 17, total = 20, *ν* = 0.85) was greater than in stage 14 oocytes (*n* = 5, total = 17, *ν* = 0.29). The most frequent abnormality in late stage 13 spindles was the presence of long, narrow poles (Fig. 2H,I). Late stage 13 spindles of *klp10A P mut*/+ heterozygous oocytes (23.5±5.1 µm, *n* = 20) differed significantly from wild type (*t* = −2.78, *d.f.* = 19, *P* = 0.01) in length, but stage 14 spindles (16.3±6.1 µm, *n* = 17) did not (Fig. 2L). These results indicate that *klp10A* knockdown and mutant effects are more severe in late stage 13 than stage 14 oocyte spindles.

### *klp10A* knockdown causes oocyte spindle mispositioning and formation of abnormal cortical microtubule asters and aggregates

**Fig. 3.**
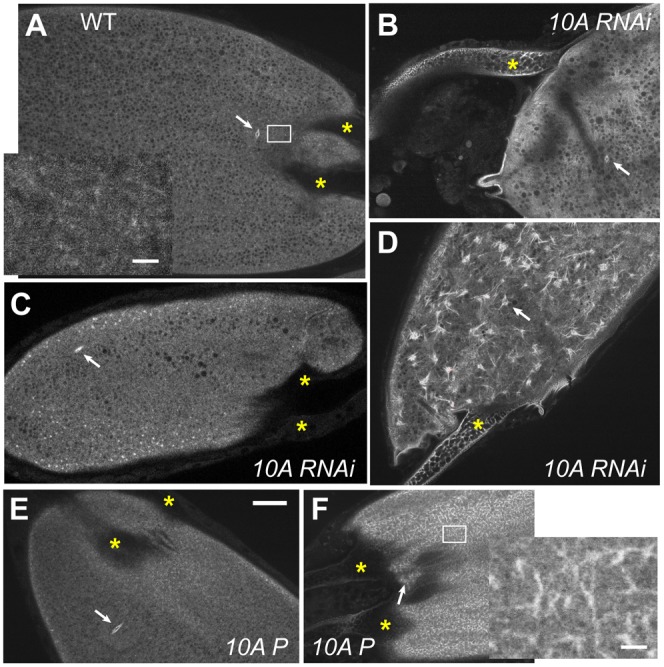
*klp10A RNAi* knockdown and a P element mutant affect oocyte MI spindle positioning and cortical microtubules. (A) Wild-type *eb1-gfp* oocyte with normally positioned MI spindle (arrow) at the base of the dorsal appendages (asterisks); cortical microtubules appear as wisplike filaments in the ooplasm (inset). (B,C) *klp10A RNAi* knockdown oocytes labeled with EB1-GFP show MI spindles displaced to the oocyte ventral side (B, arrow) or far from the dorsal appendages (asterisks), near the oocyte posterior end (C, arrow). (D) The cortical region of *klp10A RNAi* knockdown oocytes is frequently decorated with abnormal microtubule asters (arrow). (E,F) *klp10A P mut* oocytes labeled with EB1-GFP frequently showed the MI spindle (E, arrow) slightly misplaced to the side of the dorsal appendages (asterisks) or bright microtubule aggregates in the cortical region (F, inset) obscuring the spindle (F, arrow). Scale bar: 30 µm; insets: 3 µm.

We examined spindle position and cortical microtubules in live oocytes from wild-type and *klp10A RNAi* knockdown females, and *klp10A P mut line #50*/+ heterozygous females. In wild type, the bipolar MI spindle is positioned near the base of the appendages on the dorsal side of the oocyte (Fig. 3A) or close to the side of the dorsal appendages (*n* = 17, total = 17). Most of the *klp10A RNAi* knockdown spindles were present in their normal position at the base or slightly to the side of the dorsal appendages (*n* = 33, total = 47, *ν* = 0.70). However, a significant number (*n* = 14, total = 47, *ν* = 0.30) were mispositioned to the side of the oocyte, on the ventral surface (Fig. 3B), or close to the posterior end (Fig. 3C), displaced by as much as ∼200 µm or more from their normal position. Spindle positioning in the *klp10A P mut* heterozygous oocytes was closer to wild type than the *klp10A RNAi* knockdown oocytes, but a small number of spindles were mispositioned to the side of the oocyte (Fig. 3E) or behind the dorsal appendages (*n* = 9, total = 35, *ν* = 0.26). Several *klp10A RNAi* knockdown and *klp10A P mut* heterozygous oocytes showed an otherwise normal spindle with one long pole that extended to the cortex. We also observed stage 11 or 12 *klp10A RNAi* knockdown oocytes with germinal vesicles that were widely displaced from their normal position at the base of the dorsal appendages. These observations indicate that spindle mispositioning may be occurring in two ways: by early defects in germinal vesicle positioning and later defects in spindle pole anchoring to cortical microtubules after bipolar spindle formation. *klp10A RNAi* knockdown oocyte spindles were also observed that were positioned vertically rather than parallel to the oocyte cortex (*n* = 9, total = 58, *ν* = 0.16), resembling the MI spindle orientation after oocyte activation (Endow and Komma, 1997).

In wild-type *eb1-gfp* oocytes, cortical microtubules are labeled at their plus ends by EB1-GFP puncta and are barely visible as thin, wispy fibers caused by EB1 labeling along their length (*n* = 21, total = 21) (Fig. 3A, inset); this is consistent with the recent observation that EB1 binds to the lattice of growing and shrinking microtubules in the cell periphery (Currie et al., 2011). Many of the *klp10A* knockdown and mutant oocytes, however, showed cortical microtubules that were brightly decorated with EB1-GFP. The *klp10A RNAi* knockdown oocytes showed several abnormal cortical microtubule phenotypes, often within the same oocyte. The most common included a high frequency of small or large (≳0.6 µm) particles that resulted in a bright cortical region (*n* = 41, total = 91, *ν* = 0.45) or more severe effects that caused the cortical microtubules to appear as fibers, aggregates, networks, or large asters (*n* = 55, total = 91, *ν* = 0.60) (Fig. 3D). The *klp10A P mut* heterozygous oocytes showed similar mutant phenotypes, most frequently bright cortical particles (*n* = 26, total = 37, *ν* = 0.70), as well as more severe effects like those in the *klp10A RNAi* knockdown oocytes but at lower frequencies (*n* = 14, total = 37, *ν* = 0.38) (Fig. 3F, inset). The abnormal cortical microtubules were interpreted to be due to effects of reduced KLP10A on the dynamics of the microtubules – for example, the formation of EB1-labelled cortical microtubule particles and arrays could be caused by disruption of normal microtubule dynamics resulting from the lower levels of KLP10A in the knockdown and mutant oocytes. These observations implicate KLP10A in regulating cortical microtubule length and dynamics.

### *klp10A RNAi* knockdown does not affect microtubule growth rates

To determine the effect of *klp10A RNAi* knockdown on microtubule dynamics in the MI spindle, we performed EB1-GFP particle tracking experiments to analyze microtubule growth in the spindle. Because the *klp10A P mut*/+ effects on spindle length appeared to be more severe in late stage 13 oocytes than in mature stage 14 oocytes, we analyzed late stage 13 oocyte spindles by EB1-GFP tracking. EB1-GFP particles in contrast-enhanced spindle images (see Materials and Methods) were tracked and assigned as moving either poleward or equatorward. We observed significant numbers of particles moving in both directions (supplementary material Movies 1 and 2), consistent with previous observations (Liang et al., 2009), with a slight bias towards equatorward movement (∼60% of steps).

**Fig. 4.**
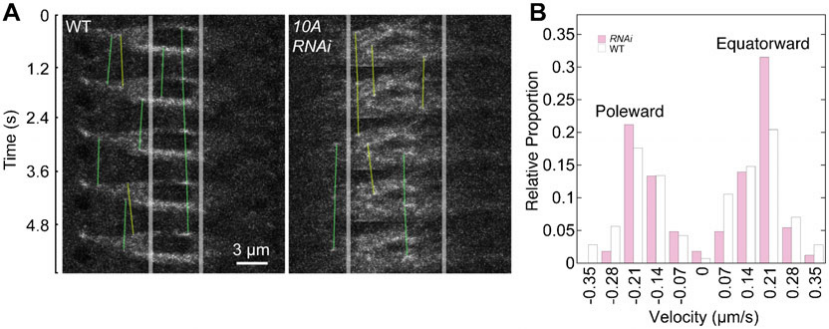
EB1-GFP particle tracking in oocyte MI spindles. (A) Kymographs of EB1-GFP particles bound to microtubule plus ends showing equatorward (yellow tracks) and poleward (green tracks) microtubule growth in wild-type and *klp10A RNAi* knockdown spindles. Images are contrast-enhanced (see Materials and Methods). The smallest particles are 1 pixel in length (0.100 µm) and are assumed to represent individual microtubule plus ends. (B) Histogram of EB1-GFP particle velocities, showing that the peak values are the same for wild-type (white bars) and *klp10A RNAi* knockdown spindles (magenta bars), both towards the pole and equator. Scale bar: 3 µm.

EB1-GFP particle velocity did not vary either with spindle length or track length, so the data for different track lengths were combined. Particle velocities were similar for the *klp10A RNAi* knockdown (equatorward, 0.183±0.007 µm/s, *n* = 97 steps; poleward, −0.167±0.006 µm/s, *n* = 68 steps; tracks, *n* = ∼26/spindle) and wild type (equatorward, 0.184±0.008 µm/s, *n* = 79 steps; poleward, −0.190±0.010 µm/s, *n* = 63 steps; tracks, *n* = ∼24/spindle) (Fig. 4A). Further, the distribution of particle velocities in the *RNAi* knockdown and wild-type spindles showed the same peak values (Fig. 4B). The mean velocities were not significantly different between the *klp10A RNAi* knockdown and wild type (*t* = −1.28, *d.f.* = 265, *P* = 0.20). These results indicate that *klp10A* knockdown does not significantly affect microtubule growth rates. However, a greater number of EB1-GFP particles in the *klp10A RNAi* knockdown spindles (*n* = 121, total = 500, *ν* = 0.242) were paused compared to wild type (*n* = 66, total = 501, *ν* = 0.132). The number of paused particles in the knockdown spindles was significantly greater than in wild type (χ^2^ = 10.4, *d.f.* = 1, *P* = 0.001), indicating that microtubules in the *klp10A RNAi* knockdown spindles spend more time in the paused state compared to wild type.

### *klp10A* knockdown slows EB1-GFP turnover in the oocyte spindle

**Fig. 5.**
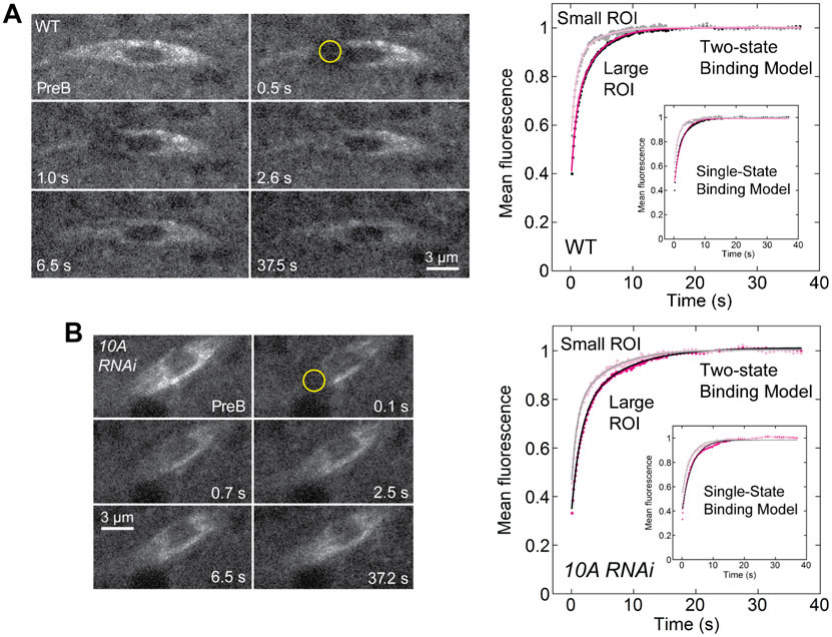
FRAP analysis of EB1-GFP binding to oocyte MI spindles. Assays of (A) wild-type or (B) *klp10A RNAi* knockdown MI spindles before photobleaching (PreB, prebleach) in a bleach spot (yellow circle) of 1 µm radius; recovery images are shown at exponentially increasing timepoints after bleaching (s, seconds). Mean normalized recovery data for wild-type (A, right) or *klp10A RNAi* knockdown spindles (B, right) were corrected for loss during imaging and fit to a two-state binding model (see Materials and Methods). *klp10A RNAi* knockdown recovery curves for small (0.5 µm radius) and large (1 µm radius) bleach spots are less steep than wild type, indicating slower dissociation of bleached EB1 and tighter EB1 binding. Insets, the same data fit to a single-state binding model, showing that the model does not capture the true kinetics of recovery for either wild-type or *klp10A RNAi* knockdown MI spindles. Scale bars: 3 µm.

In order to analyze the effects of reduced levels of KLP10A on EB1 interactions with the oocyte spindle, we performed fluorescence recovery after photobleaching (FRAP) assays on *klp10A RNAi* knockdown and wild-type oocyte MI spindles (Fig. 5A,B). We analyzed late stage 13 oocytes as above, because of the more severe effects of the *klp10A P mut* allele on spindles in late stage 13, compared to stage 14. Data from the assays (supplementary material Movies 3 and 4) using a large (1 µm radius) or small (0.5 µm radius) ROI were fit to models that yielded kinetic parameters for EB1 binding to the spindles.

Previous theoretical and empirical analysis of the relationship between *w*, the bleach spot radius; 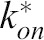, the pseudo first-order binding rate constant; *D*_f_, the diffusion constant in the absence of binding; and *k*_off_, the unbinding (or dissociation) rate constant showed that, when 
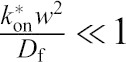
 and 
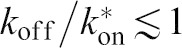
, reaction-dominant models are the most appropriate (Sprague et al., 2004). Reaction-dominant models assume that diffusion is very fast compared to both binding and the assay time, and that fluorescence recovery is dominated by dissociation of bleached protein from the cellular structure (Bulinski et al., 2001; Sprague et al., 2004). Based on these criteria and kinetic parameters derived from the full model (Sprague et al., 2004), we first tried a single-state reaction-dominant model, which postulates that recovery is dominated by a single binding state. The model was fit concurrently to the large and small ROI data points to provide additional confidence in the derived kinetic parameters (Hallen et al., 2008). The model gave good fits, but the general shape of the fits deviated somewhat from the data: fluorescence recovery in the model was too slow in early phases, and too fast in later phases (Fig. 5A,B, plot insets). The existence of these visually different phases suggested that recovery might be better modeled by including a second binding state. A two-state binding model gave visually better fits (Fig. 5A,B). This model posits that fluorescence recovery is dominated by two states, a fast diffusional binding state and a slower binding state.

The kinetic parameters derived from the curve fits of the two-state binding dominant model showed slower EB1-GFP turnover in the *klp10A RNAi* knockdown spindles compared to wild type. The *RNAi* knockdown spindles showed ∼35–40% slower unbinding (*k*_off_ and *t*_1/2_) and binding (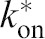) rate constants for both EB1-GFP binding states (Table 1). *C*_eq_, which is a measure of the fraction of bound protein at equilibrium, was the same or only slightly different for the *RNAi* knockdown and wild type. These results indicate that *klp10A RNAi* knockdown slows EB1 binding and dissociation in the spindle, consistent with the hypothesis that *klp10A RNAi* knockdown reduces the frequency of microtubule catastrophes. These reduced catastrophes may result in the elongated spindles we observe in the *klp10A* knockdown and mutant oocytes.

## DISCUSSION

Kinesin-13 motors are unusual in that they bind to the microtubule lattice and diffuse rapidly to the ends, where they disassemble microtubules from their ends, rather than walking along the lattice. The motors disassemble spindle microtubules, regulating microtubule dynamics, and are thought to play a role in driving poleward chromosome movement during mitosis (Rogers et al., 2004; Rath et al., 2009). The effects of kinesin-13 motors have not been as well studied in meiosis as in mitosis. For this reason, we analyzed the effects of reduced KLP10A levels on the oocyte MI spindle and the cortical microtubule network associated with the MI spindle.

### Fertility is reduced in *klp10A RNAi* knockdown and mutant oocytes

We analyzed oocytes with KLP10A levels reduced by *RNAi* or a *P* element insertion. *klp10A RNAi* knockdown females are sterile and produce oocytes with severe spindle defects and abnormal cortical microtubules, indicating that KLP10A is an essential protein during oogenesis. The *klp10A P mut* allele was only examined in heterozygous females carrying a wild-type *klp10A* allele, where the effects on oocytes are assumed to be much less severe than in homozygous females. KLP10A levels in *RNAi* knockdown ovaries were ∼45% and those in the *klp10A P mut*/+ were ∼60% of wild type, indicating that *klp10A* may be haplo-insufficient. The *klp10A P mut* allele showed reduced fertility and mutant effects on MI spindle length and cortical microtubule organization in heterozygous females, demonstrating that *klp10A* is haplo-insufficient. These findings indicate that oocytes are highly sensitive to reduced levels of KLP10A, consistent with its proposed role in regulating microtubule dynamics (Rogers et al., 2004; Mennella et al., 2005; Zou et al., 2008).

The low level of chromosome mis-segregation produced by heterozygous *klp10A P mut*/*FM7i* females indicates that nondisjunction occurs in the stock – if nondisjunction occurs during both meiotic divisions, it is expected to produce a low frequency of homozygous *klp10A P mut* females; their absence implies that homozygous females are inviable. The *klp10A RNAi* knockdown females were sterile, consistent with the severe effects on oocyte spindles and cortical microtubules that they exhibited, again indicating that oogenesis is highly sensitive to KLP10A levels.

### Spindles are elongated in *klp10A* knockdown and mutant oocytes

Both the *klp10A RNAi* knockdown and *klp10A P* element-induced mutant oocytes that we analyzed showed striking effects on spindle length. *klp10A RNAi* knockdown spindles were significantly longer than wild type in late stage 13, as well as stage 14. By contrast, *klp10A P mut* heterozygous oocyte spindles were longer in late stage 13, but appeared similar to wild type in length and overall shape in stage 14. These observations imply that KLP10A activity is more critical during the late stages of MI spindle assembly than in the mature bipolar spindle, which is arrested in metaphase I. Previous studies showed an effect of *klp10A* knockdown and mutants on spindle length in oocytes (Zou et al., 2008; Radford et al., 2012) and concluded that *klp10A* regulates spindle length, but did not provide information on KLP10A activity in different stages of spindle assembly. Our findings highlight the proposed role of KLP10A in regulating spindle length during division. This has also been observed for MCAK/Kif2C – a human kinesin-13 – which results in excessively long spindles when cellular levels are reduced by *siRNA* (Domnitz et al., 2012).

The mature stage 14 *Drosophila* oocyte MI spindle is shorter and more compact than in late stage 13, with well-defined spindle poles (Fig. 2B). Notably, *klp10A RNAi* knockdown spindles do not shorten from late stage 13 to 14, unlike wild type, where the length decrease is ∼20% – unexpectedly, the average length of *klp10A RNAi* knockdown stage 14 spindles is at least as long or slightly longer than late stage 13 spindles. Because the *klp10A RNAi* knockdown spindles do not shorten as in wild type, *klp10A* may have a role in shortening and reorganization of microtubules to form the mature MI spindle. The longer stage 14 spindles are presumably not seen in oocytes produced by *klp10A P mut*/+ heterozygous females because they express enough KLP10A from their wild-type allele to remodel the longer spindles observed in late stage 13 into normal-appearing mature stage 14 spindles.

Previous studies on a dominant-negative *klp10A* mutant showed that spindles in mutant oocytes were shorter than normal, rather than longer, as expected if wild-type KLP10A regulates spindle length by disassembling microtubules (Zou et al., 2008). The shorter-than-normal spindles were interpreted to imply that KLP10A could stabilize rather than destabilize spindle microtubules, regulating spindle length or, alternatively, that KLP10A could disrupt normal spindle assembly when mutant (Zou et al., 2008). Importantly, we observe here that reduced levels of KLP10A in oocytes produced by females heterozygous for *klp10A P mut* and a wild-type allele have a more pronounced effect on late stage 13 than stage 14 spindles (Fig. 2L), implying that KLP10A activity is more critical in earlier stages of oogenesis than in mature oocytes. Consistent with this observation, we note that many of the *klp10A NT* mutant spindles in the previous report (figure S3 of Zou et al., 2008) resemble wild-type MI spindles still undergoing assembly (Fig. 1C) (Zou et al., 2008). Taken together, it seems plausible that the dominant-negative *klp10A NT* mutant disrupts normal MI spindle assembly in the early stages of oogenesis (Sköld et al., 2005), prolonging early spindle assembly, resulting in the short spindles observed in *klp10A NT* mutant oocytes. This is consistent with a report by others of the effects of reduced levels of Kif2C on mitotic spindles. *kif2C siRNA* was observed to produce monopolar spindles in human cells (Tanenbaum et al., 2009), disrupting a pathway for normal bipolar spindle assembly. Despite the apparent differences in the effects on spindle length, the overall conclusion that KLP10A plays a role in oocyte spindle length regulation (Zou et al., 2008) is supported by the analysis of *klp10A* knockdown and loss-of-function mutants reported here and by others (Radford et al., 2012).

### *klp10A* affects spindle positioning and cortical microtubule dynamics

Spindles in both the *klp10A RNAi* knockdown and *klp10A P mut* heterozygous oocytes were frequently observed to be displaced from their normal position at the base of the dorsal appendages. In the *klp10A RNAi* knockdown oocytes, the spindles were sometimes as far away as the posterior end of the oocyte, as much as ∼200 µm or more from their normal position near the base of the dorsal appendages. Mispositioning of the oocyte germinal vesicle as early as stage 9 has been observed previously in oocytes in which dynein and kinesin-1 microtubule motors are perturbed, and has been interpreted to be dependent on these motors and attachment to the oocyte cortical microtubules (Januschke et al., 2002). Because we sometimes observed the germinal vesicle to be misplaced in early stage (≤stage 12) oocytes as well as mature stage 14 oocytes, the spindle mispositioning may be occurring by two mechanisms: mispositioning of the germinal vesicle in early stage oocytes and mispositioning of the bipolar spindle after its formation. The existence of the latter mechanism is evidenced by otherwise normal spindles with one pole stretched and extended to the cortex, as if the spindle were being displaced, which we observed in both the *klp10A RNAi* knockdown and *klp10A P mut* heterozygous oocytes. In addition, a significant number of the *klp10A RNAi* knockdown oocyte spindles appeared to be almost vertical to the cortex, as if the spindle rotation that normally occurs after oocyte activation had occurred prematurely (Endow and Komma, 1997). This is presumably caused by detachment of the spindle from the cortex, enabling the spindle to reorient vertically into a position where it can complete the meiosis I and II divisions. We concluded previously that KLP10A plays a role in anchoring the oocyte MI spindle to the cortex, based on the more vertical tilt of the spindles relative to the cortex in dominant-negative *klp10A* mutant oocytes (Zou et al., 2008).

Cortical microtubules were also observed to be abnormal at a high frequency in *klp10A* knockdown and mutant oocytes. Many *klp10A RNAi* knockdown oocytes showed large microtubule aggregates and asters, and abnormal microtubule fibers and networks were also observed in *klp10A P mut* heterozygous oocytes. The abnormal cortical microtubules were interpreted to be caused by disruption of cortical microtubule dynamics. The mechanism by which reduced KLP10A levels cause these abnormal microtubule structures to form is presumed to be reduced microtubule disassembly, based on our findings for reduced KLP10A effects on oocyte spindles and the *in vitro* activity of other kinesin-13 proteins (Walczak et al., 1996; Desai et al., 1999; Hunter et al., 2003). The effects on cortical microtubules most likely contribute to the spindle positioning defects of the knockdown and mutant oocytes.

Although the association of EB1 with the abnormal cortical microtubules implies that the microtubules are dynamic, given that EB1 is typically thought to bind only to polymerizing microtubule plus ends, we observe the abnormal cortical microtubules to undergo small, if any changes over periods of several minutes, indicating that they are probably not highly dynamic. We interpret the abnormal cortical structures to arise by the same mechanism as the elongated oocyte spindles that we observe – disruption of microtubule dynamics caused by reduced KLP10A, which reduces the frequency of microtubule catastrophes. This is consistent with the stability of the abnormal cortical microtubules over periods of several minutes or more.

Decoration of the abnormal cortical structures by EB1 can be explained by recent studies that show EB1 binding to the lattice as well as the plus ends of growing and shrinking microtubules in the cell periphery (Currie et al., 2011); we also detect EB1 lattice binding to cortical microtubules in wild-type oocytes, making them appear as faint wisplike filaments. In addition to binding to dynamic microtubules, EB1 has been reported to bind to the lattice of taxol-stabilized microtubules (Alberico et al., 2013). We further predict, based on our observations of an increase in paused microtubules and slower EB1 binding and unbinding in the *klp10A RNAi* knockdown spindles, that KLP10A also has a role in EB1 unbinding from cortical microtubules in oocytes. This is consistent with the observation by others that KLP10A remains associated with microtubule plus ends after EB1 dissociates in interphase cells (Mennella et al., 2005). Knockdown of KLP10A would therefore not only result in the formation of the abnormal cortical microtubule structures by reducing the catastrophe rate, but also in EB1 binding once they are formed. This would account for the formation of the abnormal EB1-decorated cortical microtubule aggregates and asters in *klp10A RNAi* and mutant oocytes.

### *klp10A* does not alter microtubule growth rates

The longer than normal spindles that we observe in the *klp10A RNAi* knockdown oocytes and *klp10A P mut* heterozygous oocytes could be caused by increased microtubule growth rates or decreased microtubule disassembly, including catastrophes, or both. Kinesin-13 motors have been implicated in disassembling microtubules from either end *in vitro* (Desai et al., 1999), affecting microtubule dynamics (Walczak et al., 1996; Hunter et al., 2003). We tracked late stage 13 oocyte spindle microtubules labeled with EB1-GFP at their plus ends to determine the effects of *klp10A RNAi* knockdown on microtubule growth.

EB1-GFP particle tracking showed that the rate at which spindle microtubules grow is unaffected by *klp10A RNAi* knockdown, despite the severe effects of *RNAi* knockdown on spindle length and shape. This supports the idea that the mechanism by which the *klp10A* knockdown and mutant cause elongated spindles does not involve a change in microtubule growth rates, but rather a decrease in microtubule disassembly, as expected from the microtubule depolymerization activity of kinesin-13 proteins *in vitro*.

Early studies showed that depletion of kinesin-13 XKCM1 did not affect the rate of microtubule growth or shrinkage in extracts, but did result in a 4-fold decrease in the frequency of catastrophes (Walczak et al., 1996). The mechanism by which microtubule catastrophes occur is not fully understood. Although textbook models invoke loss of a GTP cap (Alberts et al., 2007), this hypothetical cap has not yet been directly visualized. Recent studies show that the kinetics of microtubule catastrophes *in vitro* is a multi-step process that is regulated by kinesin-8 and kinesin-13 motors, which are hypothesized to act as catastrophe factors (Gardner et al., 2011). In particular, kinesin-13 MCAK/Kif2C has been proposed to convert microtubule catastrophes from a multi-step process to a single-step stochastic process (Gardner et al., 2011). This is consistent with our findings that reduced levels of the kinesin-13 KLP10A result in increased spindle length that we infer to be caused by an overall effect on microtubule disassembly, including decreased microtubule catastrophes.

### *klp10A* affects EB1 turnover

The question of how KLP10A affects EB1 binding to spindle microtubules has not been addressed in previous studies. This information is likely to be important in understanding the overall effects of KLP10A in regulating spindle length, but is difficult to obtain because it requires measuring binding constants *in vivo*. We used FRAP in these studies to analyze EB1 binding interactions with wild-type and *klp10A RNAi* knockdown oocyte MI spindles. FRAP has not been widely used to study protein binding interactions with cellular structures because exact mathematical solutions have not yet been obtained for photobleaching and recovery using high numerical aperture objectives like those typically used for these assays when performed by confocal microscopy. However, in situations where binding interactions dominate during the recovery phase, general models have been reported that can yield informative kinetic parameters for protein binding to cellular structures (Bulinski et al., 2001; Sprague et al., 2004). In these situations, the kinetic parameters estimated from reaction-dominant models provide reliable values that can be used to infer the kinetics of binding interactions (Hallen et al., 2008). Using these methods, we have previously shown that microtubule dynamics and growth rates are similar at the poles and equator of *Drosophila* oocyte MI spindles, consistent with microtubules of mixed polarity (Liang et al., 2009).

The effects of KLP10A on EB1 binding have not been reported previously, although EB1 has been shown in previous studies to target KLP10A to microtubule ends in interphase cells but not in mitotic spindles (Mennella et al., 2005). We show here that reduced levels of KLP10A in the oocyte MI spindle slow EB1 turnover, affecting both binding and unbinding of EB1 to spindle microtubules. The slower EB1 turnover rates in the knockdown oocyte spindles that we report could be caused by lower frequencies of microtubule catastrophes, if EB1 binding and dissociation are coupled to microtubule catastrophes. This effect, which has been postulated before from studies in interphase *Drosophila* S2 cells (Mennella et al., 2005), could result in the elongated spindles that we observe in the *klp10A* knockdown and mutant oocytes. We also observe an increased number of paused EB1 particles in the *klp10A RNAi* knockdown oocyte MI spindles, which may indicate microtubules pausing during the transition from growth to catastrophe. The reduced levels of KLP10A in the *klp10A RNAi* knockdown oocyte spindles could delay catastrophe, lengthening the time that microtubules spend in the paused state. An increased number of paused microtubules in *klp10A RNAi* knockdown S2 interphase cells has been observed previously (Mennella et al., 2005); at the same time, *klp10A RNAi* knockdown significantly reduced the catastrophe frequency in interphase cells. Our results indicate that increased microtubule pauses during growth also occur in *Drosophila* oocyte MI spindles, indicating that they could be linked to a decrease in microtubule catastrophe frequency. A decrease in microtubule catastrophes is consistent with both the slowed EB1 turnover that we observe in FRAP assays of the *klp10A RNAi* knockdown oocyte spindles and the increased number of paused EB1-GFP particles in the knockdown spindles.

### Concluding remarks

The results we describe here demonstrate that KLP10A functions to regulate spindle length and also affects cortical microtubule organization and spindle anchoring to the cortex. They further show that, importantly, KLP10A affects both binding and unbinding of EB1 to microtubule plus ends, which has not been reported before for a kinesin-13 motor. This implies that KLP10A may affect microtubule catastrophes in both the MI spindle and cortical microtubules, as has been described previously for interphase microtubules (Mennella et al., 2005). The interactions between KLP10A and EB1, and the mechanisms by which they occur have not yet been explained. Studies that shed light on these mechanisms will be critically important for understanding microtubule dynamics and how changes in microtubule dynamics are affected by kinesin-13 motor interactions with EB1.

## Supplementary Material

Supplementary Material
