# Supplementary material for: The kinesin-13 KLP10A motor regulates oocyte spindle length and affects EB1 binding without altering microtubule growth rates
Source: Biol Open. 2014 Jun 6;3(7):561–70. doi: 10.1242/bio.20148276 (PMC4154291; doi:10.1242/bio.20148276)
Supplement: Supplementary Material [file supp_3_7_561__index.html]

The kinesin-13 KLP10A motor regulates oocyte spindle length and affects EB1 binding without altering microtubule growth rates — Supplementary Material 

# The kinesin-13 KLP10A motor regulates oocyte spindle length and affects EB1 binding without altering microtubule growth rates

## bio.20148276 Supplementary Material

**Files in this Data Supplement:**

- Supplementary Material - Kevin K. Do et al. doi: 10.1242/bio.20148276
- Movie 1 - **Movie 1. EB1-GFP-labeled wild-type oocyte MI spindle**. EB1-GFP particles move both poleward and equatorward in the spindle. Images are contrast-enhanced.
- Movie 2 - **Movie 2. EB1-GFP-labeled *klp10A RNAi* knockdown oocyte spindle**. EB1-GFP particles move both poleward and equatorward in the spindle. Images are contrast-enhanced.
- Movie 3 - **Movie 3. FRAP assay of a wild-type *eb1-gfp* oocyte MI spindle**.
- Movie 4 - **Movie 4. FRAP assay of an *eb1-gfp; klp10A RNAi* knockdown oocyte MI spindle**.
