# Supplementary material for: The kinesin-13 KLP10A motor regulates oocyte spindle length and affects EB1 binding without altering microtubule growth rates
Source: Biol Open. 2014 Jun 6;3(7):561–70. doi: 10.1242/bio.20148276 (PMC4154291; doi:10.1242/bio.20148276)
Supplement: Supplementary Material [file supp_bio.20148276_bio.20148276-s1.pdf]

Biology Open (2014) 000, 1–10 doi:10.1242/bio.20148276

Supplementary Material
Kevin K. Do et al. doi: 10.1242/bio.20148276

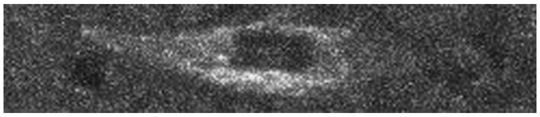

Movie 1. EB1-GFP-labeled wild-type oocyte MI spindle. EB1-GFP particles move both poleward and equatorward in the spindle. Images are contrast-enhanced.

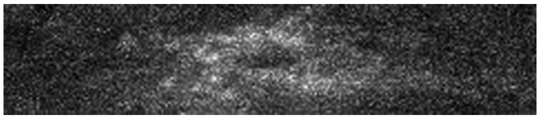

Movie 2. EB1-GFP-labeled *klp10A RNAi* knockdown oocyte spindle. EB1-GFP particles move both poleward and equatorward in the spindle. Images are contrast-enhanced.

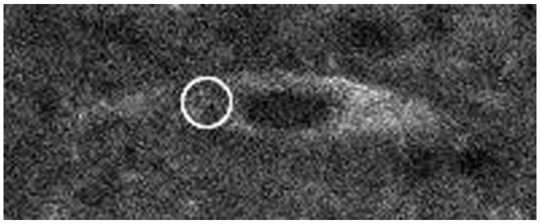

Movie 3. FRAP assay of a wild-type *eb1-gfp* oocyte MI spindle.

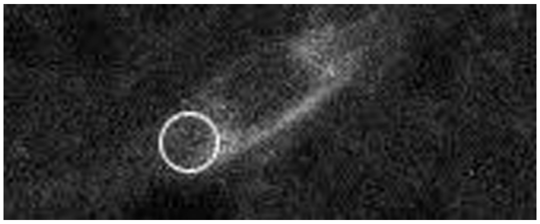

Movie 4. FRAP assay of an *eb1-gfp; klp10A RNAi* knockdown oocyte MI spindle.
